# Inhibitory control and mood in relation to psychological resilience: an ecological momentary assessment study

**DOI:** 10.1038/s41598-023-40242-1

**Published:** 2023-08-12

**Authors:** Mor Nahum, Rachel-Tzofia Sinvani, Anat Afek, Rina Ben Avraham, Joshua T. Jordan, Mattan S. Ben Shachar, Ariel Ben Yehuda, Noa Berezin Cohen, Alex Davidov, Yafit Gilboa

**Affiliations:** 1 Faculty of Medicine, School of Occupational Therapy, The Hebrew University, Jerusalem, Israel; 2 Department of Psychology, Dominican University of California, San Rafael, CA USA; 3 Ramat Gan, Israel; 4 Department of Health and Well-Being, Medical Corps, Israel Defense Forces (IDF), Ramat Gan, Israel; 5 “Shalvata” Mental Health Center, “Clalit” Health Services, Hod-Hasharon, Israel; 6 Mental Health Section, Medical Services Center, Israel Defense Forces (IDF), Ramat Gan, Israel

**Keywords:** Cognitive neuroscience, Emotion, Neuroscience

## Abstract

Psychological resilience, the ability to adapt to adversity, is theorized to rely on intact inhibitory control (IC) mechanisms, which underlie one’s ability to maintain goal-directed behavior by inhibiting prepotent responses. However, no study to date has explored daily fluctuations of IC performance in relation to resilience. Here, we examined the association between IC and mood measured daily in relation to psychological resilience in young adults in a stressful situation. Baseline resilience was obtained from 144 female and male soldiers during their basic combat training. Then, participants completed an ecological momentary assessment protocol, in which they reported their momentary mood and completed a short IC assessment twice/day for 2 weeks. A hierarchical linear modeling analysis revealed that psychological resilience moderated the relationship between momentary IC and momentary mood, such that better IC was associated with better mood only for those with higher, but not lower, self-reported psychological resilience at baseline. These results show that psychological resilience is manifested in the everyday association between IC and mood. Furthermore, they lend important support to cognitive models of resilience and may have significant contribution to our understanding of resilient behavior in real life.

Trial Registration: MOH_2018-0-13_002451.

## Introduction

Psychological or mental resilience is broadly defined as one’s ability to successfully adapt to trauma, adversity, or other significant stressors during life^1^. The ability to “bounce back” from a stressful or chronic event further involves coping with one’s emotions given the adverse situation. Studies have not just highlighted the effects of adversities on one’s well-being, but further identified multiple psychosocial traits associated with resilience, including optimism, active coping, high self-efficacy, cognitive reappraisal and greater emotion regulation^2^. In addition to individual factors, life and environmental circumstances contribute to one’s capacity to overcome adversity and related trauma^3^. However, while according to some definitions of resilience it is a fixed individual trait or a pre-disposition, others suggest that the individual properties that define resilience may vary over time^1^. In addition, better mental resilience was shown to be associated with good mental health^4^, while reduced resilience is associated with increased risk for disturbances in mental health, which may lead to depression, anxiety, and post-traumatic stress disorder (PTSD)^5^. As such, studying the factors which contribute to resilience is expected to further elucidate the potential effects of adverse life events on mental well-being.

Several recent studies suggested a central role for cognitive control—the mechanisms underlying our goal-directed behavior^6^—in mental resilience^7–9^. Among the cognitive control components, inhibitory control (IC), which underlies one’s ability to overcome an ongoing or a prepotent response and to ignore irrelevant information^10^, has been implicated as a key contributor to mental resilience^11–14^. Specifically, IC determines one’s behavior patterns, and therefore supports adaptive daily functioning^15^. Such inhibitory regulation of fundamental cognitive processes supports self-regulation and facilitates the expression of adaptive behaviors in stressful and adverse situations, hence directly contributing to resilience^16^. Accordingly, when a cherished goal is threatened, strong inhibitory control mechanisms help prioritize information related to this goal over irrelevant information, allowing one to achieve this goal despite difficulties or adversity^17^.

In line with this notion, it has been shown that better IC is associated with better resilience to potential interruptions^18,19^, with better control over emotional reactivity^20^, and with memory suppression following trauma^21^. In addition, resilience following childhood adversity was associated with enhanced inhibitory control abilities over task-irrelevant negative information^10^, and impairments in inhibitory control during emotional tasks was suggested to increase the risk for depression^13,22^.

The mechanism by which IC supports resilience is still not well understood, but may involve the exertion of control over emotional reactions^23^. Specifically, stronger IC activation may reduce emotional reactivity to negative stimuli and support the use of adaptive cognitive reappraisal strategies^8,24^. Indeed, individuals who exhibit better internal emotion regulation abilities are more likely to display resilience against adversity, compared with those with lower emotion regulation ability^25^. Moreover, the use of more adaptive emotion regulation strategies, such as reappraisal, is associated with greater IC, which is required to effectively respond to threat^26–28^. Overall, the literature to date suggests that mental resilience is dependent on intact IC mechanisms, which in turn allow for control over emotional responses. This theorized mechanism is the focus of the current study.

Despite views delineating resilience as a dynamic and interactive, rather than a static process^29^ most studies to date have used a one-time, retrospective measurement of cognitive and emotional states in relation to resilience^12,30^. However, such one-time lab-based measurements are limited in their ability to accurately characterize day-to-day variations in cognitive and emotional constructs^31^. Ecological momentary assessment (EMA) has been employed in recent years to effectively measure variations in affective and cognitive states in ecological, real-life situations^32,33^. Using EMA, data can be collected in the natural environment and repeatedly across multiple time points, allowing for more accurate representation of momentary changes^33–35^. Indeed, daily positive affect was found to be associated with lower depression and anxiety symptoms, through the enhancement of psychological resilience^33,36^. Moreover, EMA studies found that large variability in daily mood reporting is a significant predictor of mental health status^33,34,37,38^. Finally, higher mood variability over time was suggested reflect high emotional reactivity, combined with a lack of regulatory control that prevents the emotions from returning to their baseline level^39^.

However, no study to date, to our knowledge, has examined the momentary association between IC and mood in relation to resilience. The few EMA studies that did examine potential coupling between everyday mood fluctuations and changes in cognition—not in relation to resilience—show conflicting results^40,41^. For example, studies conducted by Von stuum found no associations between changes in mood and concurrent cognitive function changes, suggesting that momentary experience of positive or negative mood state does not interfere with one’s thinking capacity^41,42^. In contrast, other studies found that negative affect may be cognitively costly^43,44^, while positive affect may boost cognitive performance^45,46^.

Here, we aimed to examine the association between IC and emotional states in daily life in relation to psychological resilience during a stressful life situation. To accomplish this goal, we used obtained EMA of self-reported emotion and IC performance during a short Go-NoGo (GNG) task from male and female soldiers during their Basic Combat Training (BCT) in the Israeli Defense Forces (IDF). This design allowed us to examine whether momentary assessment of IC in relation to emotion is distinguished by different resilience characteristics. Furthermore, it allowed us to study the mechanisms of resilience during real-life stressful circumstances—BCT—in which resilience is most needed or pronounced. The exposure to stressful life events can further intensify the differences between those that have higher vs. lower levels of resilience^47^, and is therefore expected to provide a deeper understanding of its contributing factors^48^. Given the theorized association between IC, emotion, and resilience, we hypothesized here that individuals who are more resilient would show stronger association between immediate IC and emotional state under a stressful situation, compared to those with lower levels of resilience.

## Results

A total of N = 144 participants were included in the study. Participants were primarily female (n = 88, 61.1%) and between the ages of 19–21 (see Table 1 for sample characteristics). Baseline resilience was inversely associated with baseline mood (Pearson r = − 0.30, *P* < 0.001), such that those with higher resilience had overall better mood (lower IMS-12 scores); However, it was not associated with baseline IC (Pearson r = − 0.01, *P* = 0.927), nor with mood variability or with IC variability (*Ps* > 0.123). In addition, baseline IC was not associated with baseline mood (Pearson r = − 0.073, *P* = 0.52). Age at baseline was not associated with any study measures (all Pearson r < 0.16, all Ps > 0.168).

**Table 1:** Demographic and clinical characteristics of the sample (N = 144).

Variable	M (SD)	Range
Sex (female; n, %)	88 (61.1%)	
Age (years)	19.07 (0.58)	18.10–21.59
Immediate Mood Scale (IMS-12)^a^	− 67.16 (12.42)	− 91 to − 23
Baseline IC (GNG target accuracy)^a,b^	0.61 (0.19)	0.25–0.95
Resilience (Stress-resilience total score)	13.43 (3.75)	2–20

^a^Mean estimate based on LMM.

^b^See Fig. 2

### EMA data

Participants completed in total 1,153 assessment points (Mean [M] = 7.49, standard deviation [SD] = 2.95) across 2002 days, for an overall compliance rate of 57.6%. Although this indicates a large proportion of missing data, this rate is still within the range of other studies^49,50^. See also the “Analysis or Missing Data” section in the “Methods”.

Unconditional ICCs were 0.309 (95% CI 0.245, 0.380) for the IMS-12 and 0.529 (95% CI 0.441, 0.615) for GNG, respectively. This suggests that approximately 69.1% of the variance in mood and 47.1% of the variance in IC can be attributed to within-person variability. There was no change in GNG performance over time (*P* = 0.21), nor in mood over time (*P* = 0.35). These analyses are detailed in Supplementary Material 1. In addition, average IC performance across the EMA sessions was weakly correlated with baseline resilience (r = 0.157, *P* = 0.029).

### Primary analysis

**Figure 1:**
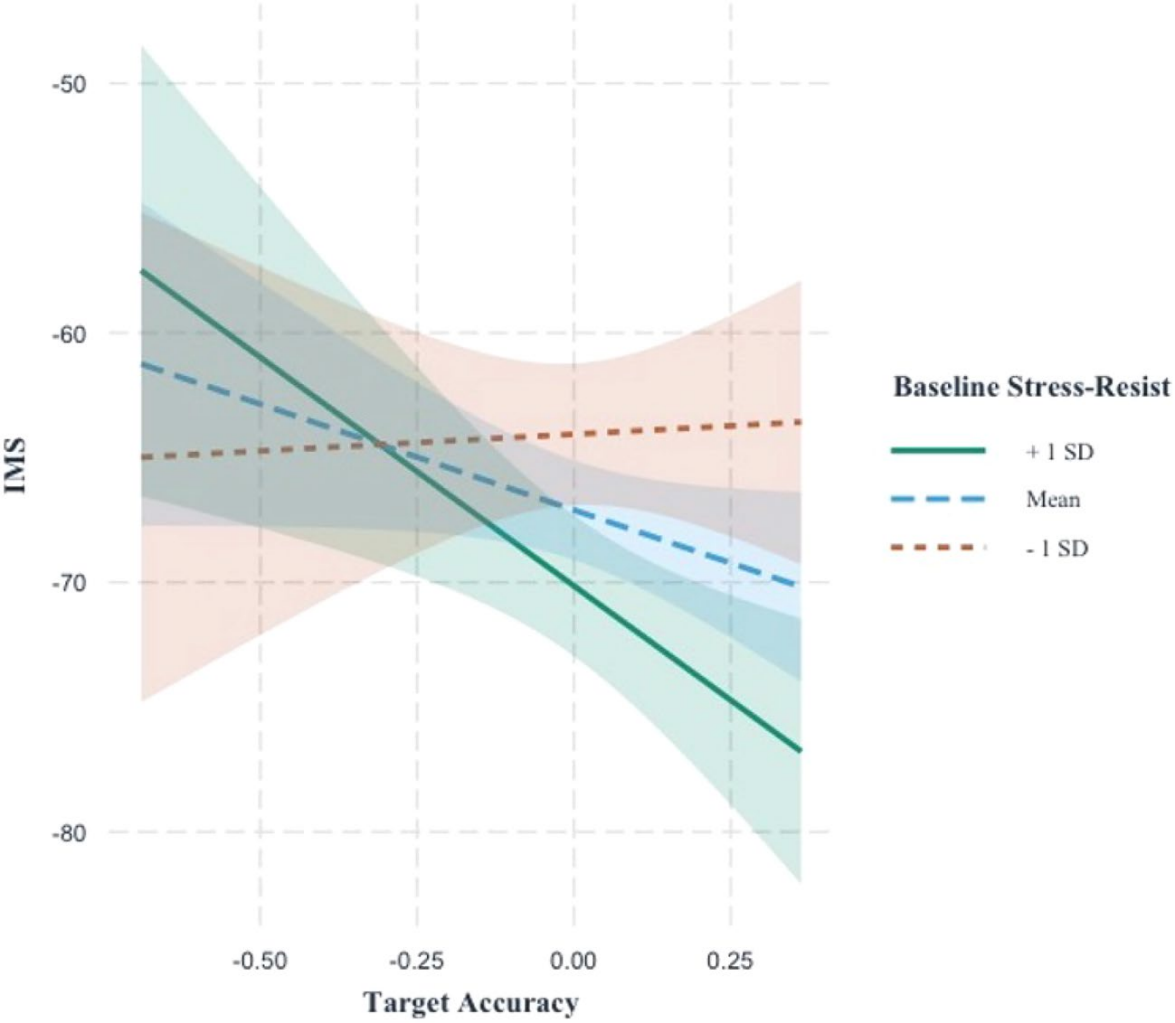
Baseline resilience moderates the relationship between IC (target accuracy for the GNG task, x-axis) and mood (IMS-12 total score, y-axis). The association between IC and mood is significant for higher baseline resilience (green line) but not for lower resilience (dashed red line). Note that lower IMS-12 score indicate better momentary mood.

Results from the LMMs can be found in Table 2. In the first step, IC had a small, inverse association with IMS-12 (*sr* = 0.08, |*t*|= 2.06, *P* = 0.04), such that better IC was associated with better mood. Model fit improved with the inclusion of baseline resilience as a moderator of IC and mood (second step), although this improvement was small per conventional guidelines (${f}^{2}$= 0.04)^51^. IC was no longer independently associated with mood (*sr* = 0.07, |*t*|= 1.87, *P* = 0.06); however, there was a significant interaction between IC and baseline resilience (*sr* = 0.19, |*t*|= 2.03, *P* = 0.04). An examination of simple slopes (see Fig. 1) revealed that there was no association between IC and mood when baseline resilience was low (defined as -1 SD; *b* = 1.34, |*t*|= 0.19, *P* = 0.85), or when baseline resilience was average (defined as the mean; *b* = − 8.49, |*t*|= 1.87, *P* = 0.061), but that there is an association between IC and mood when baseline resilience is high (defined as + 1 SD; *b* = − 18.32, |*t*|= 2.89, *P* = 0.004).

**Table 2:** Linear mixed-effects models testing the association between cognitive control and baseline resilience on mood.

Variable	Step 1				Step 2			
	*B* (SE)	|*t*|	*P*	*sr*^a^	*B* (SE)	|*t*|	*P*	*sr*^a^
Intercept	− 67.16 (1.04)	64.61	< .001	–	− 67.11 (1.02)	66.00	< .001	–
IC (GNG target accuracy)	− 9.32 (4.53)	2.06	.04	0.08	− 8.51 (4.55)	1.87	.06	0.07
Baseline resilience					− 0.80 (0.27)	2.96	.004	0.18
IC (GNG target accuracy) × Baseline resilience					− 2.60 (1.28)	2.03	.043	0.19
Marginal *R*^2^/Conditional *R*^2^	0.006/0.331				0.044/0.338			

^a^*sr* semi-partial correlation.

### Exploratory analyses

We conducted two additional exploratory analyses. First, we examined sex effects in the data, applying the model separately to female and male participants. Note that this analysis should be interpreted with caution, as the study was not powered for this effect. Second, we examined the potential effect of motivation on the relationship between mood and IC. These analyses are detailed in Supplementary Material 2.

## Discussion

In this study, we examined the manifestation of psychological resilience in everyday life in a group of 144 young adults under stress, during their basic combat training in the military. Better resilience at baseline was associated with better mood at baseline, but not with better baseline IC, nor with mood or IC variability. Using EMA of IC and of mood, we found that for those with higher levels of self-reported resilience, higher momentary IC was associated with better momentary mood. However, no such association was found for those with medium or lower levels of resilience.

To our knowledge, this study is the first to show that higher levels of resilience are associated with specific associations between IC and mood in daily life. As such, it validates and broadens the conceptualizations of current models of resilience, which suggest a central role for cognitive control—and mainly IC processes—in contributing to resilient outcomes in the face of adversity or during stressful life situations^7,9^. Specifically, the model by Parsons and colleagues suggests that resilience relies on one’s ability to apply appropriate cognitive control processing in each situation, using an overarching mapping system, which guides the application of information processing based on situations and perceived needs^7^. Previous studies have indeed provided validation for this and related models, using a one-time measurement in the lab, which showed the expected association between IC and resilience^15,52^. Similarly, literature related to resilient outcomes following childhood trauma or malfunctioning further suggests that those with better psychological resilience are those who can better activate frontoparietal areas and that are able to exert better emotion regulation strategies^52,53^. Here, we provide for the first time the ecological validation of this assertion, by showing that the momentary level of IC is related to one’s current emotional state only in those who are more resilient.

Our results are also in line with imaging studies, which show the association between increased mental resilience and prefrontal control over limbic areas; this suggests that IC mechanisms are closely related to mood and resilience. Furthermore, it has also been shown that stress resistance depends on control-induced activation of the ventral medial prefrontal cortex (vmPFC) over the brainstem and limbic system. As a result of control experience, vmPFC circuitry is activated by later uncontrollable stressors, which inhibits stress-responsive limbic and brainstem structures, thereby resulting in stressor resistance. This proactive stressor resistance generalizes across different stressors and may be involved in determining individual differences in reactions to adversity^54^. For example, a study by Admon and colleagues which examined resilience in Israeli soldiers found that lower amygdala reactivity pre-deployment predicted lower PTSD symptom levels post-deployment, as did greater increase in hippocampal-vmPFC functional coupling from pre- to post-deployment^55^. A recent study by Demers and colleagues included a sample of healthy adults with and without a history of childhood trauma and found that across the entire sample, better adaptive functioning in everyday contexts was associated with increased IC over negatively-valenced distractors and greater prefrontal (right frontal pole) activation. The authors concluded that resilience in the face of early adversity is associated with enhanced inhibition of task-irrelevant negative information^10^. Our results are consistent with this conclusion.

The results of the current study further substantiate the definition of resilience into a real-life stressful situation, showing that the momentary association between IC and mood underlies self-reported resilience. As such, these results provide the first ecological validation of the definition of resilience, which relies on the IC ability to inhibit variations and momentary mood reductions. Since cognitive abilities, including IC, have been shown to fluctuate over time given different levels of fatigue, stress and external conditions^56,57^, a one-time measurement of IC may not provide the full picture of IC abilities, stressing the need for a repeated, ecological assessment of IC and its association with momentary mood provides additional information.

While no previous study, to our knowledge, has examined the association between momentary IC and momentary mood in relation to mental resilience, our results are still in line with some of the previous literature on EMA which shows association between IC and emotion in clinical and healthy samples. For example, a study by Smith and colleagues^58^ examined daily IC and momentary mood in relation to binge eating behavior in women with eating disorders. To that end, they tested the associations between intra-individual variability in self-regulatory capacity with negative affect. The authors found that among those who reported regular compensatory behaviors, binge eating was more likely on days with lower momentary negative affect when food-related IC was better. Conversely, lower momentary negative affect was related to lower likelihood of binge eating on days with poorer IC. However, since IC was examined only once per day, it is unclear whether negative affect impairs momentary ICs’ capacity or vice versa^58^.

Other studies have attempted to assess the momentary association between other cognitive control abilities and emotion, yielding mixed results. For example, in a few studies conducted in a group of young adults it was found that on days with more reported negative affect and reduced motivation, performance on a working memory (WM) task was also reduced (see also^43–45^). Recent studies by Von Stumm showed no effect of mood on WM performance among healthy adults^41^. Similarly, Small and colleagues found no associations between inter-subject variability of self-reported depressed mood and performance on WM tasks; however, they did find a link between self-reported average scores of fatigue and cognitive tests in a small sample of cancer survivors^59^. A potential account for this lack of association between momentary cognitive control and mood given our current results is that baseline resilience has not been considered. Thus, it could be that for participants with higher levels of psychological resilience, such associations would have been found. Future studies are needed to further elucidate the link between cognitive control—and IC in particular—and momentary emotional states in ecological setting in relation to psychological resilience, in both clinical and non-clinical samples.

The exploratory analyses conducted here revealed a significant sex effect: momentary IC was associated with momentary mood in female participants with higher levels of resilience, but not in male participants. This finding should be interpreted with great caution, given the lack of power to perform reliable analyses on these data. Still, it is unlikely that the sex differences in our study can be accounted for by differences in the type or severity of adversity, since all participants were in the same environment. A possible account is that the resilience construct is not sex sensitive. Indeed, men typically score higher on measures of resilience compared to women since existing conceptualizations of resilience do not reflect the way that sex roles, social expectations, perceptions, and the environment interact to differentially shape women’s and men’s experiences of adversity and their responses to it^60^. Future studies with larger samples should be conducted to validate these exploratory analyses and further examine the everyday manifestation of resilience in females compared to males.

This study has a few limitations that should be considered in future studies of ecological manifestation of IC, emotion, and resilience. A main limitation is that due to the field conditions of the study, the number of EMA samples per participant was rather low and may limit the generalizability of our findings. Although the number of samples was sufficient for the analyses conducted, denser sampling, as was done is other studies (e.g.,^37,61^) is advised and may have significantly strengthened the analytical approach and the validity of the findings. In addition, denser sampling during the day would have allowed us to predict mood from previous IC level, which should be accomplished in future studies. The use of a mood assessment scale which includes separate items for positive and negative mood would have also provided richer understanding of the effects of IC on current mood. Moreover, our sample, of young adults from a distinct mixed-gender unit of the IDF, is relatively homogeneous in terms of age and nationality, and includes twice as many female participants. This may limit the generalizability of the findings and calls for replication studies in broader samples.

The findings of the current study, which used “in the field” measurement of daily IC and mood, further substantiate the definition of resilience into a real-life stressful situation, showing association of IC with resilience, as well as momentary association between IC and mood underlies self-reported resilience. Our results provide the first ecological validation of the definition of resilience, which relies on the IC ability to inhibit variations and momentary mood reductions. Consequently, these results lend valuable support to cognitive models of psychological resilience and may contribute to our understanding of resilient behavior in the real world.

There are two significant implications for these results. First, they emphasize the importance of including smartphone-based EMA tools in studying cognition and emotion. The increasing use of smartphones and related mobile technology over the last decade^38^ makes it easier to deliver EMA assessments remotely and provide significant data with high ecological validity. As noted previously, the use of mobile assessments allows the integration of multiple sources of contextual and cognitive data and hence may improve the precision of the data collected^35^. In addition, the results may have implications for interventions which aim to strengthen psychological resilience under stressful situations. Given the malleability of resilience, it is considered a potential intervention target which may improve one’s ability to cope with stressful situations and adversities^8^. Our results suggest that incorporating ecological momentary interventions (EMIs^31^) to improve or build resilience may help bridge the gap in current mental health care for youth and young adults by enabling better access to interventions “in each moment” and in an appropriate context in daily life^62^. Thus, novel interventions may incorporate EMI with existing interventions to achieve better resilience amidst stress in young adults. Future studies should examine the utility of such interventions on resilience.

## Methods

### Participants

The study was approved by the Institutional Review Board (IRB) of the IDF medical corps and was performed in accordance with the relevant guidelines and regulations. A convenience sample of IDF soldiers (n = 154) was recruited for the study. Participants were from two recruiting cycles of the border defense infantry battalions, who were studied during their basic combat training (BCT), between April 2018 and October 2019. Data collection was conducted at the recruits’ military base in the Southern part of Israel. The border defense infantry recruits both male and female soldiers who undergo similar training together. All participants provided written informed consent before engaging in any study-related activities, and none received monetary compensation for their participation.

Of note, the study reported here is part of a larger, randomized controlled trial (RCT) study, in which we examined the effects of computerized cognitive training among soldiers^63^. The larger study was powered to detect between-group effect size of Cohen's d of 0.5 (see, e.g.,^64^), with a power of 0.8 and alpha error probability of 0.05. Here, we report data from N = 144 participants who had EMA data.

### Study design

Participants completed baseline assessments (t0) and then completed 2 weeks of EMA, which included momentary mood reporting and a real-time IC task. The study then continued with repetition of the baseline assessments (t1), followed by 2 weeks of cognitive training. The results reported here refer to a subset of the data collected during t0 (psychological resilience) and the 2 weeks of EMA. Results from other parts of the study are reported elsewhere^15,63,65–67^.

### Measures

#### Baseline psychological resilience (t0)

We used the Hebrew version of the self-assessed resilience scale^68^. This 5-item scale asks participants to rate their ability to cope with stress on a 4-point Likert scale on each of the 5 items (0 = low ability, 4 = excellent ability). Items include questions regarding their ability to “keep calm and think of the right thing to do in a crisis,” “manage stress,” “try new approaches if old ones don’t work,” “get along with people when you have to,” and “keep your sense of humor in tense situations”. The total score for the scale is derived from the sum of scores of all five items, and ranges between 0 and 20, with higher scores reflecting better self-perceived psychological resilience. The scale had an acceptable internal consistency in our sample (Cronbach’s alpha = 0.732). Previous studies reported slightly higher internal consistency for this scale (Cronbach’s alpha of 0.86–0.89)^69^.

#### Ecological momentary assessment (EMA)

EMA data of mood and IC was collected during a 2 weeks period following t0 using the Moodify app^34^. The app was installed on participant’s mobile phones, and they were asked to provide, twice/day during weekdays (i.e., Sunday–Thursday), a momentary mood reporting and then complete a short IC task. Since the soldiers participating in the study did not have their mobile phones with them, their commanders were requested by the study staff to provide them with their phones twice/day, once in the morning and once in the evening, to allow them to complete the EMA tasks. Due to their varying schedule during BCT, we provided a relatively broad time window of 4.5 h in the morning (6 am–10:30 am) and in the evening (6 pm–10:30 pm) for completion of the EMA assessments. Participants could complete the EMA assessments only once during each time window and assessments were unavailable in times outside these two time windows. Daily reminders were sent to the commanders by the study staff, reminding them to give the soldiers their phones during the relevant time window. Participants were contacted by study staff in case they missed several consecutive assessments, to help with any technical issues they encountered. The overall completion time for each EMA session was ~3 min.

Momentary mood reporting was collected using the Immediate Mood Scale (IMS-12;^34^), a 12-item measure which was developed to assess the dynamic components of mood. The IMS-12 scale prompts participants to rate their current mood state on a continuum using 12 items (e.g., happy-sad, distracted-focused, sleep-alert, fearful-fearless), each with a 7-point Likert scale. For each item, an integer score between 1 and 7 was derived. The total score for this scale is the sum of the scores on all 12 items. To be consistent with other scales assessing mental health status (e.g., PHQ-9, GAD-7), the total score is multiplied by -1, such that where higher scores reflect worse (i.e., more negative) mood states. The generalizability coefficients of the IMS-12 (i.e., generalization of daily measurements across observation) in our sample was Rc = 0.948.

**Figure 2:**
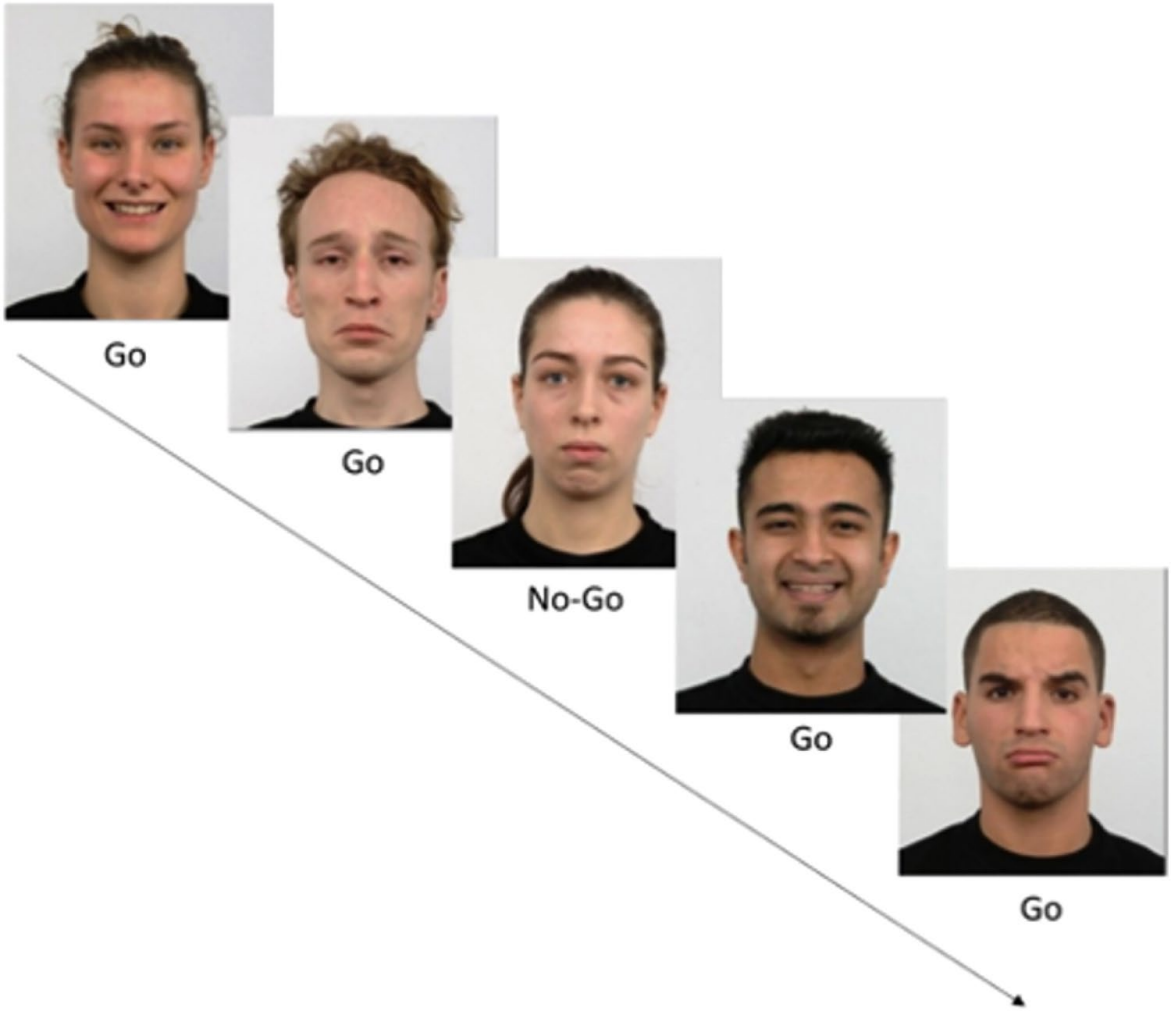
Example of the emotional Go/No-Go task used in the study. Images showing facial expressions appear sequentially on the screen. The user should respond quickly to emotional faces (either happy or sad foil images/Go; 80% of trials) and withhold from responding to rare neutral faces (target stimuli/No-Go; 20% of trials). Images were taken from the KDEF image set. Written consent for publication of human identity revealing images was obtained from the creators of the KDEF set. The task included 100 trials.

Momentary IC was assessed using an abbreviated version of an affective GNG task, which provides an objective measure of IC, as it measures prepotent response inhibition (the ability to withhold or cancel a speeded motor response), which is considered a central component of IC^70^. The task included a total of 100 trials and takes ∼2.5 min to complete. On each trial, participants are asked to tap a button appearing on the screen as fast as possible whenever a frequent (80% of the time) foil picture was presented, and to withhold response to rare (20% of the time) target pictures. The task began with the presentation of a “start” button at the center of the screen. Once the user clicked on the ‘start’ button, the pictures were presented sequentially, each for a 1000 ms, with an inter-stimulus interval (ISI) of either 500, 1000, and 1000 or 1500 or 2000 ms (randomly chosen for each trial). The presented stimuli were pictures of emotional expressions taken from the KDEF set. Target pictures were of neutral facial expressions, while foil stimuli were of emotional facial expressions (either sad or happy faces). Of the 80 foil images, 40 included faces with happy expressions and 40 with sad expressions. Auditory feedback was provided after each trial to indicate the correctness of response. For analysis, we derived the metric of target accuracy (accuracy in withholding on No-Go trials; range: 0–1), comprising an acceptable measure for prepotent IC, compared to commission errors^71^. The generalizability coefficients of the target accuracy metric in our sample was Rc = 0.610 (Fig. 2).

### Analysis of missing data

Due to the relatively high percentage of missing data, we examined patterns of missingness in the data in several ways^72–74^. First, we examined how the number of IC measurements, number of IMS-12 assessments and maximal study day were correlated with any of sex, age, baseline resilience, and baseline IC metrics (target/foil mean RT, SDRT, accuracy), and the IMS-12 items. Of the 69 correlations examined, only 1 was significant, but did not remain significant when applying FDR correction (all Ps > 0.69).

Second, we examined if missingness for each measure (IMS-12, mean RT, SDRT, and mean ACC) at each time point can be predicted by any of the measurements at the previous time point. This analysis can potentially allow for detection of missing not-at-random (MNAR) patterns of missingness (e.g., participants with high IMS-12 scores at time t might be less likely to report IMS at time t + 1). Here too, none of the associations was statistically or clinically significant (all FDR corrected Ps > 0.20).

We also performed a mixed-effects logistic regression to examine whether missingness at a given point in time was related to missingness at the previous time point. Applying this analysis, we found that missingness was related to missingness at the previous time point (OR = 1.43, *p* = 0.007). Since participants reported that they were often unable to comply/respond due to field conditions (lack of cellphone service, lack of access to phones due to activity), these results suggest that data was missing due to participants random inability to comply.

We conclude that the results of the missing data analysis suggest that the data was missing completely at random (MCAR). We therefore opted to use linear mixed-effects models (LMM; described in the next section) which can tolerate such patterns of missingness, allowing the use of all available data (rather than complete cases only).

### Statistical analyses

We tested whether the relationship between IC and mood varies as a function of resilience through linear mixed-effects models (LMM). For all analyses, restricted maximum likelihood (REML) was used, and Kenward-Roger degrees of freedom were implemented for small-sample adjustment^75^. Prior to running primary analyses, we examined variability in mood and IC (per the IMS-12 and GNG, respectively) via Intraclass Correlations (ICC) using an unconditional LMM. The ICC provides an estimate of the proportion of variance that can be attributed to between-person variability (1-ICC, therefore, is the proportion of variance that can be attributed to within-person variability). We further examined reliability of the EMA metrics by computing generalizability coefficients for multilevel data. These measures are similar to Cronbach’s $\alpha$, but they account for the repeated measures structure of the data and can be used to estimate reliability of change scores^76^. Finally, we made sure that reaction times (RTs) of the GNG task were withing the acceptable range and that no outlier data was included. Indeed, average RTs across EMA sessions for each participants ranged between 227 and 754 ms (mean: 543.1 ± 34.25 ms), which is within the acceptable range given the nature of the task.

We then tested two LMMs hierarchically: in the first step, IC (participant mean-centered) was included as a time-varying predictor; in the second step, resilience at baseline (group mean-centered) and its interaction with IC were included. As an exploratory analysis, we also examined whether the association between IC and baseline resilience on mood varies as a function of sex. The method proposed by Stoffel, Nakagawa, and Schielzeth^77^ was used to provide effect size estimates for all predictors via squared semi-partial correlations (${sr}^{2}$). In brief, their approach iteratively removes predictors from a model and examines the change in the variance of the linear predictor, and the difference to the full model provides an estimate of the unique variance explained by individual predictors^77^. We took the square root of these estimates for it to be consistent with the traditional correlation coefficient effect size estimate. Because REML is incompatible with traditional indices of relative model fit (such as change in the Bayesian Information Criterion), model goodness-of-fit was examined through marginal and conditional R^2^, where marginal R^2^ represents the proportion of variance explained by fixed factors and conditional R^2^ represents the proportion of variance explained by fixed and random factors^78^. Improvement in model fit (with the inclusion of baseline resilience as a moderator) was examined via Cohen’s ${f}^{2}$, where change in marginal R^2^ served as the measure of model fit^79^. Significant interaction terms were probed via simple slopes analysis. Statistical significance was evaluated at a two-sided alpha = 0.05. All analyses were conducted in R^80^ with the lme4^81^, lmerTest^82,83^, interactions^84^, psych^85^, and partR2^77^ packages.

### Supplementary Information

Supplementary Information 1.

Supplementary Information 2.

## Data Availability

The datasets generated during and/or analyzed during the current study are not publicly available due to ethical restrictions and for de-identification purposes but are available from the corresponding author on reasonable request.

## Abbreviations

BCT: Basic combat training
EMA: Ecological momentary assessment
EMI: Ecological momentary interventions
GNG: Go-NoGo
HLM: Hierarchical linear modeling
IC: Inhibitory control
ICC: Intraclass correlations
IDF: Israeli Defense Forces
IMS: Immediate mood scale
LMM: Linear mixed-effects models
PFC: Prefrontal cortex
PTSD: Post-traumatic stress disorder
REML: Restricted maximum likelihood
WM: Working memory
