# Supplementary material for: Inhibitory control and mood in relation to psychological resilience: an ecological momentary assessment study
Source: Sci Rep. 2023 Aug 12;13:13151. doi: 10.1038/s41598-023-40242-1 (PMC10423230; doi:10.1038/s41598-023-40242-1)
Supplement: Supplementary file 1 — Supplementary Information 1. [file 41598_2023_40242_MOESM1_ESM.docx]

**Supplementary Material 1**

**Analysis examining changes in mood and IC over time**

For both GNG performance (target accuracy of No-Go trials) and mood (IMS-12 total score), we have modeled a simple growth model over days. The models were fit using both random intercepts for participants and random slopes for days (per Barr et al., 2013). There were no significant changes in neither IC nor mood over days. The results are detailed below.

## IC (GNG target accuracy of No-Go trials)


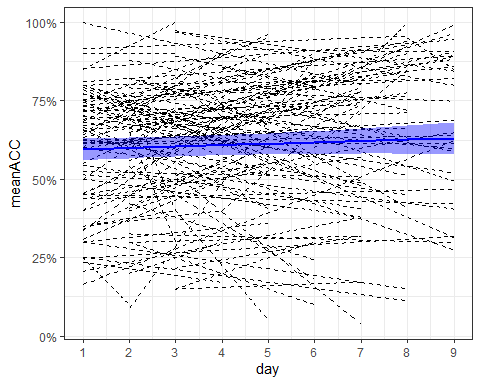


# Fixed Effects

| Parameter | Coefficient | SE | 95% CI | t | df | p |
| --- | --- | --- | --- | --- | --- | --- |
| (Intercept) | 0.590 | 0.020 | (0.551, 0.630) | 29.757 | 124.20 | < .001 |
| day | 0.004 | 0.003 | (-0.003, 0.011) | 1.249 | 84.58 | 0.215 |

# Random Effects

| Parameter | Coefficient |
| --- | --- |
| SD (Intercept: X_personal.number) | 0.155 |
| SD (day: X_personal.number) | 0.014 |
| Cor (Intercept~day: X_personal.number) | 0.176 |
| SD (Residual) | 0.153 |

## Mood (IMS-12 total score)


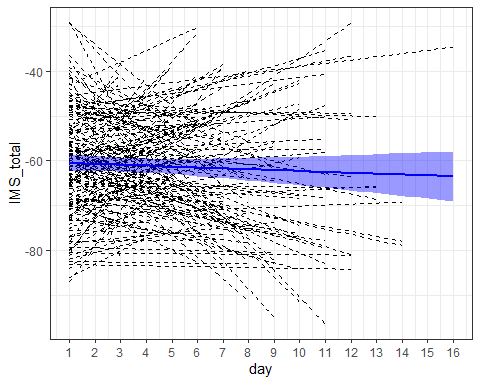


# Fixed Effects

| Parameter | Coefficient | SE | 95% CI | t | df | p |
| --- | --- | --- | --- | --- | --- | --- |
| (Intercept) | -60.301 | 1.076 | (-62.427, -58.175) | -56.057 | 147.72 | < .001 |
| day | -0.199 | 0.215 | (-0.624, 0.227) | -0.925 | 118.00 | 0.357 |

# Random Effects

| Parameter | Coefficient |
| --- | --- |
| SD (Intercept: X_personal.number) | 9.907 |
| SD (day: X_personal.number) | 1.762 |
| Cor (Intercept~day: X_personal.number) | -0.508 |
| SD (Residual) | 12.103 |
