# Supplementary material for: Inhibitory control and mood in relation to psychological resilience: an ecological momentary assessment study
Source: Sci Rep. 2023 Aug 12;13:13151. doi: 10.1038/s41598-023-40242-1 (PMC10423230; doi:10.1038/s41598-023-40242-1)
Supplement: Supplementary file 2 — Supplementary Information 2. [file 41598_2023_40242_MOESM2_ESM.docx]

### **Supplementary Material 2**

### Exploratory analyses

**Sex effects**

We exploratorily examined sex effects in the data. Males and females did not differ on baseline resilience (|*t*| = 1.12, *P* = .26, Cohen’s *d* = 0.19), mood (|*t*| = 1.21, *P* = .23, Cohen’s *d* = 0.19), or IC (|*t*| = 0.15, *P* = .88, Cohen’s *d* = 0.03). Furthermore, there was no difference in average mood or IC overall assessment points (|*t*| < 1.45, *P* > .15). Unconditional ICCs for mood differed between males (ICC = 0.509, 95% CI = 0.397, 0.620) and females (ICC = 0.236, 95% CI = 0.166, 0.324), indicating that females reported greater within-person variability in mood than males over the course of the study. Thus, exploratory models were tested with and without individual-level variability (as measured by the Root Mean Square of Successive Differences; RMSSD) as a fixed covariate. Because the pattern of differences did not change when including RMSSD as a covariate, results below are from unadjusted models.

The results are summarized in **Table S1**. Including sex as a moderator had a small improvement in overall model fit (Cohen’s $f^{2}$= 0.02). There was a significant three-way interaction between sex, IC, and baseline resilience on mood (*sr* = 0.20, |*t*| = 2.53, *P* = .012). Simple slopes analysis indicated that males did not exhibit an association between IC and mood (all *P* > .215), but females observed an association between IC and mood at average and high levels of baseline resilience (*b* = -11.20, |*t*| = 2.17, *P* = .03 and *b* = -27.88, |*t*| = 3.75, *P* < 0.001; see **Figure S1**).

**Table S1**. Exploratory Linear mixed-effects model testing the association between IC and baseline resilience on mood as a function of sex.

| **Variable** | | ***B* (SE)** | | **\|*t*\|** | | | ***P*** | | ***sr*** |
| --- | --- | --- | --- | --- | --- | --- | --- | --- | --- |
| Intercept | | -68.67 (1.66) | | 41.38 | | | < 0.001 | | -- |
| IC (GNG Target Acc) | | -6.83 (9.61) | | 0.71 | | | .48 | | 0.03 |
| Sex (Female) | | 2.62 (2.09) | | 1.255 | | | .21 | | 0.08 |
| Resilience | | -1.34 (0.44) | | 3.05 | | | **.003** | | 0.18 |
| GNG Target Acc X Sex | | -4.41 (10.91) | | 0.40 | | | .69 | | 0.08 |
| GNG Target Acc X Resilience | | 3.61 (2.86) | | 1.29 | | | .20 | | 0.18 |
| Resilience X Sex | | 0.89 (0.56) | | 1.61 | | | .11 | | 0.20 |
| GNG Target Acc X Resilience X Sex | | -8.08 (3.20) | | 2.53 | | | **.012** | | 0.20 |
| Marginal *R*^2^/Conditional *R*^2^ | 0.068/0.354 | |  | |  |  | |  |  |

Note. *sr* = semipartial correlation.


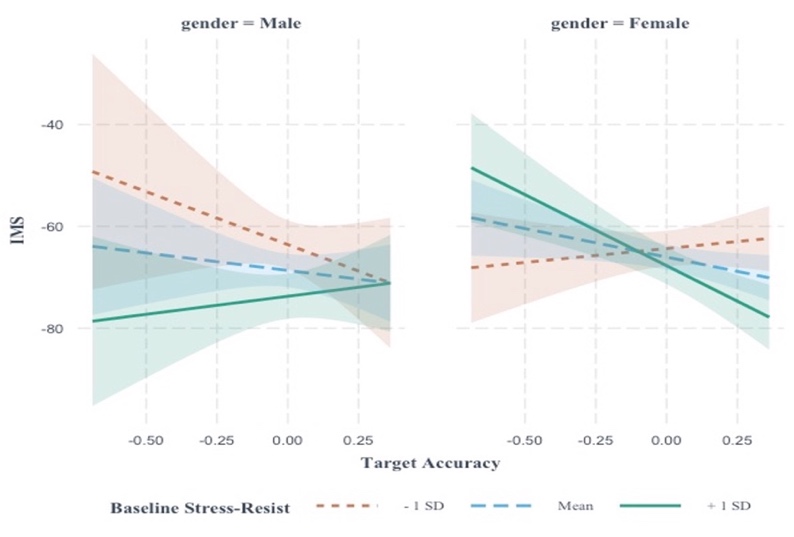


**Figure S1**. Sex moderates the relationship between baseline resilience and daily IC on daily mood.

**Motivation Effects**

We next examined whether the relationships between mood and IC were not merely a byproduct of motivation, with participants in a better mood being more motivated to perform the IC task. The IMS-12 has 2 items which are related to motivation (apathetic vs. motivated and numb vs. interested). We therefore computed the IMS total scores without these 2 items (deriving an IMS-10 total score) and examined the associations between IC and mood (IMS-10). Our results show that there is no substantial change in the effect of IMS-10 on IC (or its interaction; **Table S2**). Note that although the estimated coefficients are slightly reduced, the pattern of results remains the same, and the overall effect of IC remains significant. We therefore conclude that the relations between mood and IC are not a byproduct of increased motivation in better mood.

**Table S2**. Linear mixed-effects models testing the association between cognitive control and baseline resilience on mood (IMS-10).

| **Parameter** | **Step 1** | **Step 2** |
| --- | --- | --- |
| (Intercept) | -51.31 (0.84), p <0.001 | -51.09 (0.80), p <0.001 |
| IC (GNG target accuracy) | -7.97 (2.67), p =0.003 | -6.77 (2.64), p =0.011 |
| Baseline Resilience |  | -0.79 (0.21), p <0.001 |
| IC X Baseline Resilience |  | -1.05 (0.68), p =0.126 |
|  |  |  |
